# Osteopontin Augments M2 Microglia Response and Separates M1- and M2-Polarized Microglial Activation in Permanent Focal Cerebral Ischemia

**DOI:** 10.1155/2017/7189421

**Published:** 2017-09-20

**Authors:** Anne Ladwig, Helene Luise Walter, Jörg Hucklenbroich, Antje Willuweit, Karl-Josef Langen, Gereon Rudolph Fink, Maria Adele Rueger, Michael Schroeter

**Affiliations:** ^1^Department of Neurology, University Hospital of Cologne, Cologne, Germany; ^2^Medical Imaging Physics, Institute of Neuroscience and Medicine (INM-4), Research Centre Juelich, Juelich, Germany; ^3^Cognitive Neuroscience, Institute of Neuroscience and Medicine (INM-3), Research Centre Juelich, Juelich, Germany

## Abstract

**Background:**

Focal cerebral ischemia induces distinct neuroinflammatory processes. We recently reported the extracellular phosphor-glyco-protein osteopontin (OPN) to directly affect primary microglia *in vitro*, promoting survival while shifting their inflammatory profile towards a more neutral phenotype. We here assessed the effects of OPN on microglia after stroke *in vivo*, with focus on infarct demarcation.

**Methods:**

Animals underwent focal photothrombotic stroke and were injected intracerebroventricularly with 500 *μ*g OPN or vehicle. Immunohistochemistry assessed neuronal damage and infarct volume, neovascularisation, glial scar formation, microglial activation, and M1 and M2 polarisation.

**Results:**

After photothrombotic stroke, areas covered by M1 and M2 microglia substantially overlapped. OPN treatment reduced that overlap, with microglia appearing more spread out and additionally covering the infarct core. OPN additionally modulated the quantity of microglia subpopulations, reducing iNOS+ M1 cells while increasing M2 microglia, shifting the M1/M2 balance towards an M2 phenotype. Moreover, OPN polarized astrocytes towards the infarct.

**Conclusion:**

Microglial activation and M1 and M2 polarization have distinct but overlapping spatial patterns in permanent focal ischemia. Data suggest that OPN is involved in separating M1 and M2 subpopulations, as well as in shifting microglia polarization towards the M2 phenotype modulating beneficially inflammatory responses after focal infarction.

## 1. Introduction

Focal cerebral ischemia induces an activation of brain resident glial cells and an influx of peripheral immune cells [1]. Those inflammatory responses have been termed a “double-edged sword” since they not only serve beneficial functions, such as phagocytosis of debris and recruitment of neural stem cells, but also mediate secondary tissue damage [2]. Multiple activational states of microglia are often coined in a dichotomous way as M1 and M2 subtypes, with more proinflammatory and detrimental effects attributed to the M1 phenotype while more regulatory and protective actions attributed to M2 phenotypes [3]. We previously characterized the process by which microglia as the resident immune cells of the brain separate vital brain tissue from ischemic necrosis [4]. At the very margin of those two, the “demarcation zone” is characterized by microglia actively secreting the nerve/glial antigen 2 (NG2) into the extracellular matrix (ECM), thereby separating necrosis from vital tissue with a decisive impact on secondary tissue damage [4]. Recently, Gliem et al. reported the ECM glycoprotein osteopontin (OPN) to be involved in the demarcation of focal ischemic lesions by elongating astrocytic processes, improving astrocyte-mediated neovessel coverage [5]. Our own recent work revealed direct effects of OPN on primary microglia *in vitro*, promoting their survival while shifting their inflammatory profile towards a more neutral phenotype [6]. Although exact protein concentrations are unknown, OPN protein is virtually absent in the naïve brain, but OPN mRNA is upregulated by some 5000 times in the infarct area after focal cerebral ischemia. The absence of OPN has been shown to increase secondary neurodegeneration [7]. Based on these observations, we hypothesized that OPN would modulate the inflammatory profile of microglia after stroke *in vivo*, while potentially affecting the process of infarct demarcation as well.

## 2. Methods

### 2.1. Animals and Surgery

#### 2.1.1. Focal Ischemia Model: Photothrombosis (PT)

Adult male Fisher 344 rats (*n* = 15) weighing 200–300 g were sedated with isoflurane (2–5%), subsequently anesthetized with i.p. ketamine (75 mg/kg)/medetomidine (0.5 mg/kg) and atraumatically fixated in a stereotactic frame.

After disinfection, the scalp was longitudinally incised (10 mm) and the skull was exposed. Periost was removed leaving the bone intact. Bregma and lambda points were identified. A fiberoptic bundle of a cold light source with an aperture of 1.5 mm was placed onto the skull using a micromanipulator and adjusted to the stereotactic coordinates 2 mm posterior and 3 mm lateral from the bregma. The skull was illuminated for 20 minutes with a white light beam (150 W). During the first 2 minutes of illumination, the rats were injected with the dye rose bengal (Sigma, Munich, Germany 1%, 1 *μ*g per g bodyweight).

Following surgery, the wound was sutured and the animals were allowed to recover from anaesthesia. Afterwards, they were put back into their home cages and were given access to food and water ad libitum.

#### 2.1.2. OPN Treatment

To assess the effects of OPN in cerebral ischemia *in vivo*, one day after induction of photothrombosis, the animals were randomly assigned to the treatment groups and treated with a single intracerebroventricular (i.c.v.) injection of either 500 *μ*g recombinant OPN (R&D Systems) in 5 *μ*l saline (*n* = 8), or 5 *μ*l saline as control (placebo group, *n* = 7). To this end, each animal was put under anaesthesia with ketamine (75 mg/kg) and medetomidine (0.5 mg/kg) and injected stereotactically using the following coordinates: bregma anteroposterior −0.9 mm, mediolateral +1.4 mm, and ventrodorsal +3.8 mm.

After injection, the animals were allowed to recover from anaesthesia and were returned to their cages, where they were given access to food and water ad libitum.

Eight days post i.c.v. injection, rats were sacrificed under deep anaesthesia.

### 2.2. Histology and Immunohistochemistry

Serial coronal brain sections (slice thickness 20 *μ*m) were cut through the area of greatest infarct diameter on a cryostat (CM3050S, Leica Instruments).

To assess the patterns of inflammatory cell infiltration in MCAO and photothrombosis, brain sections were stained with a selection of antibodies against an M1- and M2-polarized microglia (Table 1). Microglia stainings were supplemented by stainings against antineuronal nuclear (NeuN) antigen to assess the extent of ischemic damage, von Willebrand factor (vWF) for the identification of (neo-)vascularisation, the astrocytic marker GFAP, and anti-NG2, an antigen recognized on oligodendrocyte and microglial precursors (Table 1).

Depending on the antibody, the sections were either fixed with 4% PFA for 15 min, 30 min at room temperature (RT), or overnight at 4°C. All following incubation steps were conducted at RT unless mentioned otherwise. After fixation, the slides were washed in 0.1 M PBS, quenched in H_2_O_2_ (only in 3,3′-diaminobenzidine (DAB) staining), washed in 0.1 M PBS again, and blocked with 5% normal goat serum, 5% normal donkey serum (Jackson Immuno Research Laboratories, Baltimore, USA), or 5% normal horse serum (Vector Laboratories, Burlingame, CA, USA) in 0.1 M PBS (with or without 0.3%Triton X) for 45 min. The sections were incubated with the primary antibody overnight at 4°C (dilutions are detailed in Table 1).

After rinsing and washing in 0.1 M PBS, the secondary antibody was applied and incubated for 30 min. Secondary antibodies used were biotinylated goat anti-rabbit IgG, biotinylated horse anti-mouse IgG, and biotinylated horse anti-goat IgG (Vector Laboratories, Burlingame, CA, USA).

For light microscopic visualization, the ABC Elite Kit (Vector Laboratories) followed by diaminobenzidine (Sigma Aldrich, Munich, Germany) as final reaction products were used.

Secondary antibodies for fluorescence microscopy were donkey anti-rabbit 488 IgG, donkey anti-goat 568 IgG, donkey-anti-mouse 488 IgG, goat-anti-mouse 488, and goat-anti rabbit 568 (Alexa Fluor, Invitrogen/Life Technologies, Carlsbad, CA, USA). For nuclear costaining, Hoechst 33342 (Life Technologies, Darmstadt, Germany) was applied for 10 min after rinsing the secondary antibody.

Representative images were taken using an inverted fluorescence phase-contrast microscope (Keyence BZ-9000E, Osaka, Japan) with 4x, 20x, and 40x objectives.

### 2.3. Image Analysis

Sequentially scanned images of the infarct area were reconstructed in the BZ-II Analyzer (Keyence, Neu-Isenburg, Germany). All measurements and evaluations were performed by a blinded evaluator (A.L.) with the open-source software ImageJ/Fiji (Rasband, W.S., ImageJ, US National Institutes of Health, Bethesda, Maryland, USA, https://imagej.nih.gov/ij/, 1997–2014). The interrater reproducibility of the threshold measurements was verified by remeasurements of randomly chosen samples by a second investigator (H.L.W.).

#### 2.3.1. Area Measurements

In order to evaluate the percentage of the infarct area covered by a certain cell type, the infarct area (delineated by NeuN staining) was marked with the polygon selections tool and cut out. Subsequently, the threshold tool was used to selectively mark the cell-like formations stained with the respective antibody and to subtract the background. The total infarct area and the remaining area were measured in *μ*m^2^, and the percentage was calculated using Microsoft Excel 2011.

#### 2.3.2. Cell Counts and Area Measurements in Specific Infarct Zones

**Figure 1:**
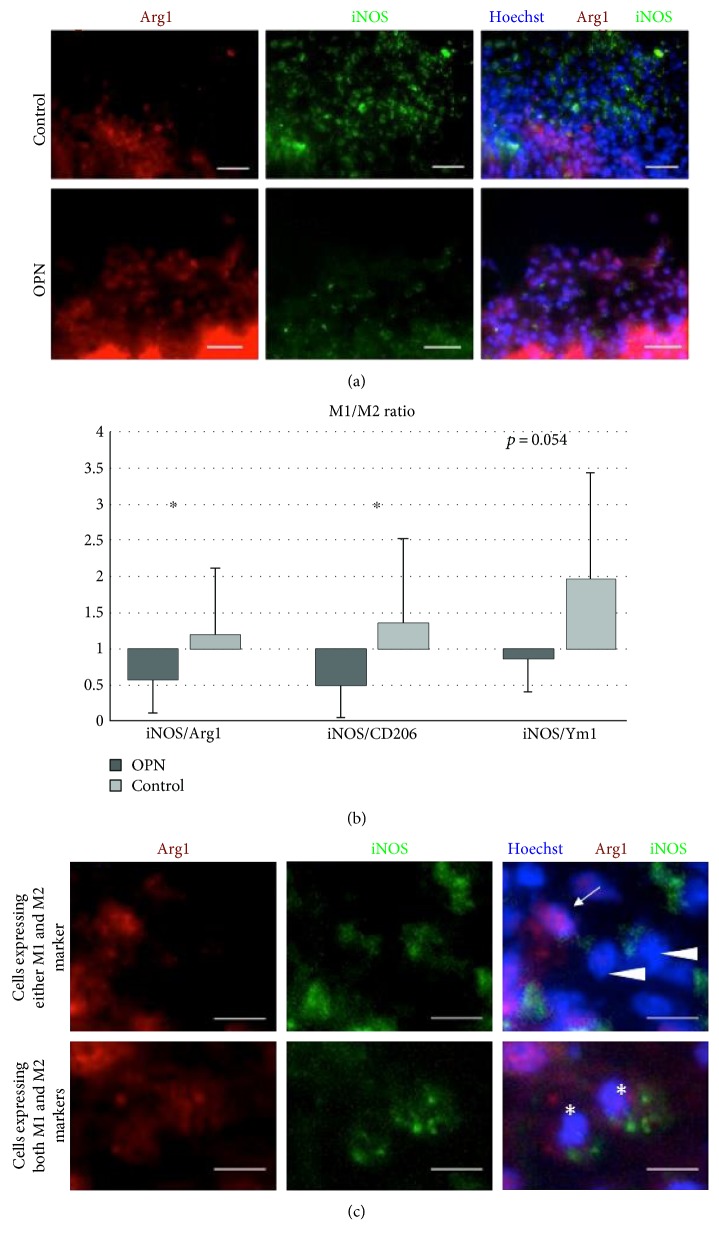
OPN treatment shifts the M1/M2 balance towards an M2 phenotype. (a) Arg1+ M2 cells (red) dominated the infarcted area in OPN-treated rats, whereas iNOS+ M1 microglia were found predominantly in vehicle-treated animals (green). (b) M1 (iNOS-expressing) microglia and M2 (Arg+, CD206+, or Ym1+) microglia in the infarcted area after photothrombosis expressed as M1/M2 ratio. Ten days after photothrombosis, the ratios of M1/M2 microglia were significantly decreased in OPN-treated animals compared to vehicle-treated controls, suggesting an increase in M2 polarization. (c) Close-up of microglia expressing either M1 (iNOS) or M2 (Arg1) markers (upper row), or expressing both markers (lower row). Scale bars represent 40 *μ*m in (a) and 10 *μ*m (c). All graphs show mean and SD; ^∗^*p* < 0.05.

To quantify the observed changes in stratification of the inflammatory cells within the infarct area (Figure 1), cell densities and areas covered by one cell type were measured within the respective infarct zone. Zones were defined according to the Iba1 staining. The densest Iba1 belt defined the infarct margin at days 8–10 after ischemia as described earlier [4]. The area between the hypocellular core and the infarct margin was labelled as the outer infarct core. The area delineating necrotic tissue from viable tissue as defined by NeuN-positive neurons was termed as the demarcation zone [4]. Outside of the infarct border laid the peri-infarct zone, which was characterized by a continuously increasing density of Iba1+ cells toward the infarct. It transitioned into the unaffected tissue. Representative fields of view (measuring 100 × 100 *μ*m) were chosen from each animal from each zone, and the area covered by the individual cell type within this 10000 *μ*m^2^ square was measured.

#### 2.3.3. Planimetry

To assess the effect of OPN on absolute and relative size of each infarct zone, the Iba1-stained slides were used, and measurements were performed using the polygonal section tool from ImageJ/Fiji.

#### 2.3.4. Generation of Pseudocolored Overlays

Adjacent images stained with all mentioned antibodies were edited with the threshold tool of ImageJ/Fiji. In a picture-editing program (Pixelmator 3.3 for Mac), those images were assigned pseudocolors and adjacent sections were aligned manually with the aid of anatomical landmarks.

#### 2.3.5. GFAP Polarization and Measurement

GFAP-positive cells, which were preserved and stained completely, were counted and assigned to a polarized or unpolarized group depending on their process alignment [5]. The processes were measured.

### 2.4. Statistical Analysis

Statistical evaluations were performed using the GraphPad Prism software. Data sets were tested for normal distribution with the D'Agostino-Pearson normality test. For normally distributed data sets, a parametric test was used (unpaired *t*-test for two samples). For unequal variances between two samples, Welch's *t*-test was assigned. A nonparametric test was used for nonnormally distributed data sets (Mann-Whitney test for two samples). *p* value was set at 0.05 for statistical significance.

## 3. Results

### 3.1. Functional Layering of Microglia Subtypes

**Figure 2:**
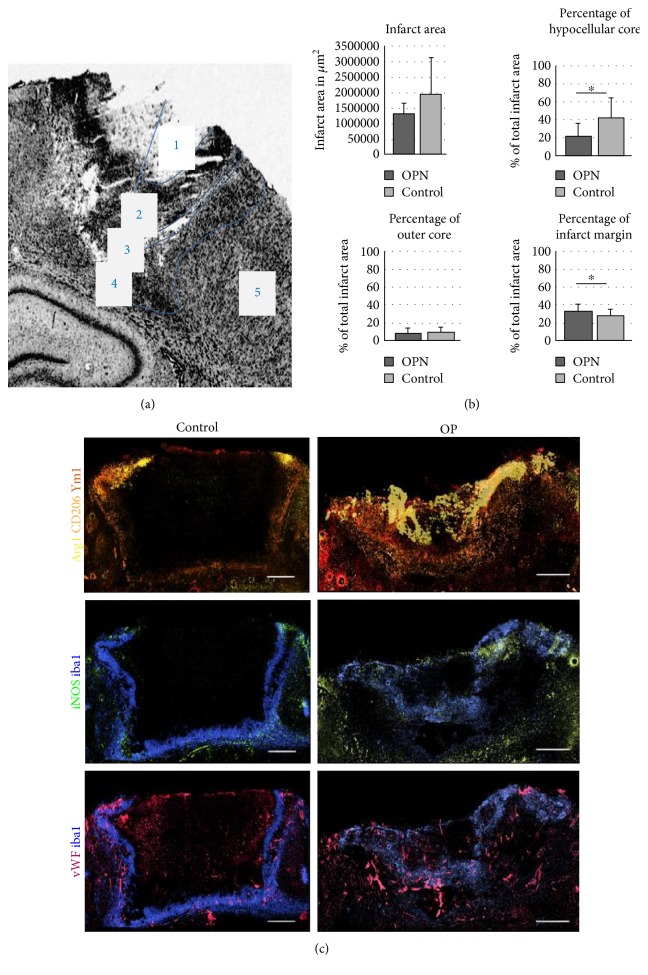
OPN increases functional layering of microglia subtypes and influences the relative size of distinct infarct subareas. (a) Cresyl violet staining of the photothrombotic infarct. The infarct area could be subdivided into five zones according to distribution pattern of inflammatory cells: hypocellular infarct core (1), cell-rich infarct margin (2), demarcation zone (3), peri-infarct zone (4), and unaffected tissue (5). (b) The size of the evaluated infarct area shows a trend towards smaller infarcts, but no significant changes under OPN treatment. The infarct layers, especially those close to the core show significant changes. The size of the hypocellular core is significantly reduced post OPN and the infarct margin is proportionally larger. (c) Displays pseudocoloured images of exemplary photothrombotic infarcts. Sections were stained for Arg1 (yellow), CD206 (orange), YM1 (red), iNOS (green), Iba1 (blue), and vWF (purple). Adjacent sections of an OPN-treated animal (right column) and a control rat (left column). Scale bars represent 300 *μ*m (c). All graphs show mean and SD; ^∗^*p* < 0.05.

Neuroinflammation helped to define multiple subareas of the infarct tissue organized in centrifugal order: hypocellular infarct core, outer infarct core, infarct margin, demarcation zone, and peri-infarct zone (Figure 2(a)). The closer to the infarct margin, the more microglia were activated. The distribution of differently polarized subtypes of microglia—M1 and M2—in and around the ischemic lesion was assessed immunohistochemically. Pseudocolors were assigned to each marker to visualize the distribution of M1- and M2-polarized microglia in OPN-treated and control brains (Figure 2(c)). In controls, neuroinflammation was confined to a comparatively thin belt around the necrotic infarct core, with the different microglial subpopulations overlaying each other to a great extent (Figure 2(c)). In OPN-treated animals, microglial subpopulations overlapped less and rather spread out into the lesion itself (Figure 2(c)). In particular, Arg1+ M2 microglia extended centripetally into the infarct core, diminishing the hypocellular part to a minimum (Figure 2(c)). Thus, OPN induced a more pronounced stratification of inflammatory cells within the infarct area.

### 3.2. Relative Sizes of Distinct Infarct Subareas

Immunohistochemical data were used to quantify the relative size of the distinct infarct subareas in control versus OPN-treated animals. After OPN injection, we observed a smaller hypocellular infarct core (*p* = 0.0045) but a wider infarct margin (*p* = 0.0407) than under control conditions (Figure 2(b)).

### 3.3. Infiltration of Microglia Subpopulations into Distinct Infarct Subareas

**Figure 3:**
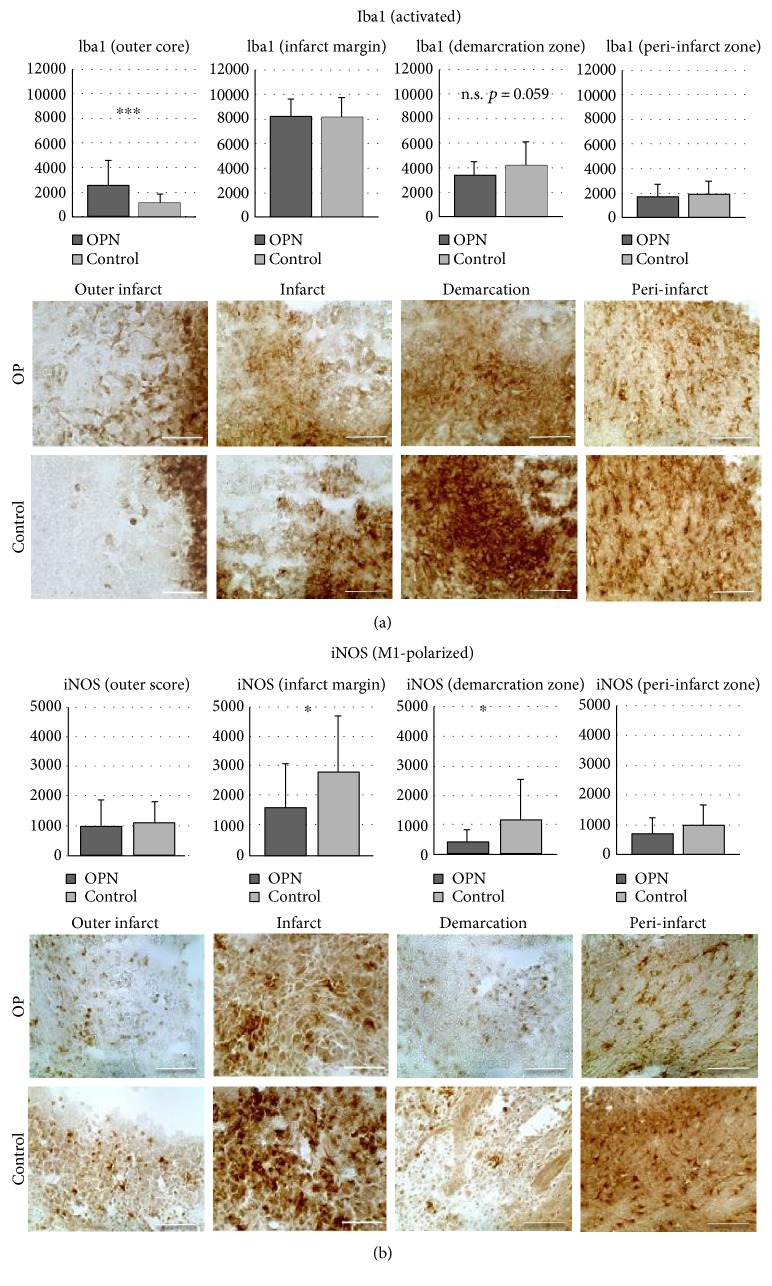
OPN modulates the infiltration of microglia subpopulations into distinct infarct subareas (part I: Iba1 and iNOS). All microglia stained with Iba1+ (a) and proinflammatory microglia stained with iNOS (b) shown in distinct subareas of the photothrombotic infarct after OPN treatment compared to control. Iba1+ cells are found elevated the outer infarct core, while iNOS+ cells (M1) are decreased in the infarct margin and the demarcation zone in OPN-treated animals. Scale bars represent 50 *μ*m. All graphs show mean and SD; ^∗^*p* < 0.05; ^∗∗∗^*p* < 0.001.

**Figure 4:**
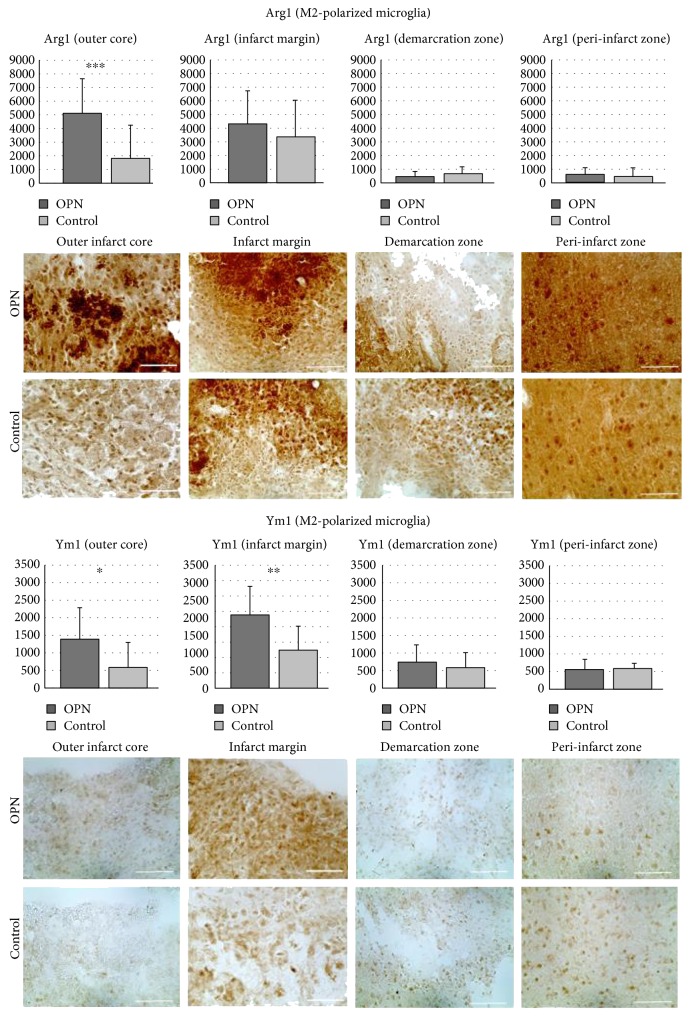
OPN modulates the infiltration of microglia subpopulations into distinct infarct subareas (part II: Arg1 and Ym1). M2 subpopulations stained with Arg1 and Ym1 in subareas of photothrombotic infarct after OPN compared to placebo treatment. Arg1+ microglia show an increase in the outer infarct core, while Ym1+ microglia are found enriched in the outer core and infarct margin. Scale bars represent 50 *μ*m. All graphs show mean and SD; ^∗^*p* < 0.05; ^∗∗^*p* < 0.01; ^∗∗∗^*p* < 0.001.

The ischemic lesion was subdivided into five distinct regions (Figure 2(a)). In the outer infarct core, general Iba1+ activated microglia were more pronounced after OPN-treatment than under control conditions (*p* = 0.0008, Figure 3(a)), and the same was observed for M2-polarized microglia (Figure 4), suggesting OPN to activate microglia and induce their M2 polarization. In parallel, and particularly in the infarct margin, expression of iNOS by M1 microglia was decreased by OPN (Figure 3(b)).

### 3.4. Ratio between M1 and M2 Microglia

Ratios between M1- and M2-polarized microglia were assessed on the basis of immunohistochemical stainings for the M1 marker iNOS and the M2 markers Arg1, CD206, and Ym1. Arg1+ M2 cells dominated the infarct in OPN-treated rats, whereas iNOS+ M1 microglia were found predominantly in control animals (Figure 1(a)). For all three combinations (iNOS/Arg1, *p* = 0.0406, iNOS/CD206, *p* = 0.0401, and iNOS/Ym1, *p* = 0.054), M1/M2 ratios were decreased following i.c.v. injection of OPN into stroke rats (Figure 1(b)). We observed not only cells expressing either M1 or M2 markers but also others expressing both markers at once (Figure 1(c)).

### 3.5. Astrocyte Polarization

**Figure 5:**
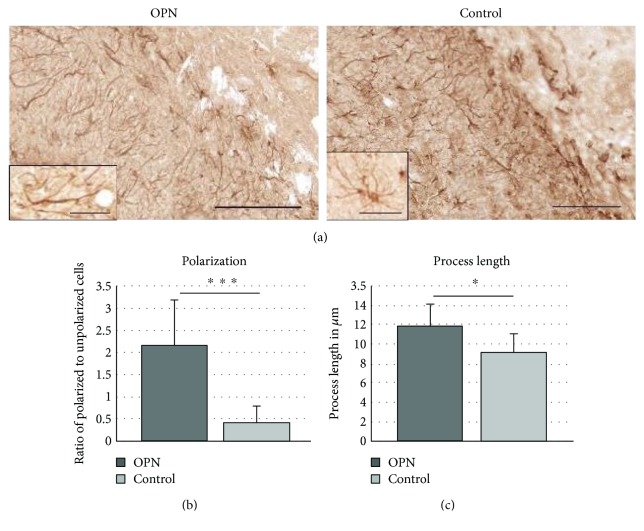
OPN polarizes astrocytes towards the infarct. (a) Infarct margin stained for GFAP showing the majority of astrocytes with processes aligned towards the infarct core in OPN-treated animals, and increased process length in OPN-treated animals. Quantification of changes in polarisation (b) and process length (c). All graphs show mean and SD. ^∗^*p* < 0.05; ^∗∗∗^*p* < 0.001.

GFAP+ astrocytes forming the glial scar around the infarct adopted a bipolar form in OPN-treated animals (Figures 5(a) and 5(b)). Polarized astrocytes were characterized by an elongated shape, pointing their processes towards the infarct core. The length of the cellular processes was significantly increased after OPN compared to controls (Figure 5(c)).

## 4. Discussion

Permanent focal cerebral ischemia elicits a profound neuroinflammatory reaction dominated by resident microglia and macrophages recruited from the blood stream. This reaction is initiated immediately after the onset of ischemia and lasts for weeks [8]. Inflammation occurring in defined spatiotemporal patterns strongly impacts on the outcome after ischemia [9–11].

### 4.1. Microglial Activation

The microglia/macrophage population is heterogeneous not only in origin but also in morphology and function. Upon activation, microglia undergo characteristic morphological changes. In a sequence of events, they shift from a resting ramified phenotype via a transient hyperramified phenotype towards a stellate and later ameboid shape. Microglial activation reflects the severity of tissue damage and may well extend beyond the site of primary tissue damage. The description of previous studies had already described two nonoverlapping subpopulations of CD4+ and CD8+ microglia/macrophages in rats, providing early evidence for heterogeneous microglia/macrophage populations in poststroke inflammation [12–15]. As described more recently, activated microglia may functionally polarize towards an M1+ or M2+ cell phenotype [3, 16, 17]. The direction of polarization is essentially influenced by the surrounding “milieu” [18, 19]. In an inflammatory setting, OPN might be a suitable candidate for establishing such a “milieu” [20]. Being secreted into the ECM by microglia, it acts through paracrine and autocrine signalling, thereby activating microglia and interacting with other cells that impact on regeneration, such as astrocytes, neural stem cells, and endothelial cells [20–22].

### 4.2. M1 And M2 Polarization

We here describe that polarization of microglia towards an M1 and or M2 phenotype could be recognized in photothrombosis, extending earlier results in focal ischemia [23]. In the photothrombotic infarct, we detected M1 microglia predominantly in the peri-infarct area at in the vicinity of the demarcation zone. M1 microglia is thought to produce oxygen species and generally thus create a toxic environment [24]. The M1 shift goes along with a switch in mitochondrial energy production from oxidative phosphorylation to anaerobic glycolysis further increasing metabolic demands, lactate acidosis, and free radical production [3]. The localization of M1 microglia with high energy demand in the peri-infarct zone reported here—and given functional connotations of M1 microglia—make these cells likely to contribute to secondary damage after focal ischemia.

Ineffective phagocytic activity of microglia goes along with the failure of containment of necrotic tissue, causing ongoing microglial activation and loss of trophic support for surrounding neurons; these processes can cause further bystander damage, as is known from neurodegenerative diseases [25–27]. M2 polarization is suggested to be the more “benign” activation state, accelerating removal of debris and repairing damaged tissue. We here observed M2 microglia within the necrotic infarct core area and in close proximity to sites of neovascularisation.

Of note, in our study, microglial activation did not necessarily go along with either M1 or M2 polarization. An essential proportion of Iba1+, microglia exhibited stellate or ameboid morphology but expressed neither M1- nor M2-typical markers. This is in accordance with the hypothesis of multistep und tightly regulated microglial activation resulting in multiple functional and metabolic subtypes [28].

### 4.3. OPN Effects

We found a decreased ratio of M1 and M2, implying a shift towards M2 microglia cells 10 days after the induction of ischemia. We here investigated whether the M1 to M2 shift was a fluent transition, or represented two distinct nonoverlapping populations that lack the ability to switch. While iNOS is the typical marker for M1 microglia, Arginase1 signifies the functional antagonist of iNOS, which is the reason why those two enzymes are the favoured markers for M1 and M2 polarization. In the infarct margin, the distinction between M1 and M2 (Arg1, iNOS) was less strict than expected from the single cell level. Many cells appeared to coexpress M1 and M2 markers iNOS and Arg1, respectively, signals indicating a concept of a continuous phenotypical shift rather than distinct microglial subpopulations expressing one or the other [3, 18, 29, 30]. We found an inversely proportional correlation between M2 markers (Arg1 [*p* = 0.0322, *r*^2^ = 0.1773], CD206, [*p* = 0.0022, *r*^2^ = 0.3132]) and infarct size, being compatible with the earlier notion of protective functions of M2 microglia [31, 32]. However, the study design did not allow proving a causative relationship between increased M2 polarization and stroke protection.

Data suggest that better-defined layering of microglia/macrophage subtypes after stroke is a surrogate for beneficially modifying poststroke inflammation processes. Our results are in line with the assumption that M1/M2 dichotomy is not absolute, as predicted by findings about the Th1/Th2 dichotomy of lymphocyte activation [32]. Rather, M1/M2 polarization represents a relative dynamic state along a spectrum of activational conditions, and activated microglia may even express both or none of the polarization markers (Figure 1(c)). However, a clear separation of M1 and M2 cells may be beneficial for the outcome after stroke (Figure 2()).

Previous studies also described enhanced neovascularisation in the vascular stem cell niche as well as an increased migration of stem cells out of their niche after ischemia [33, 34]. We have recently reported an increased migration of stem cells after stroke when treated with OPN i.c.v. [35]. Accordingly, OPN might be able to mediate between microglia and stem cells. In line with those previous results, the current study demonstrated an increased vascularization of the infarcted area in OPN-treated animals. Moreover, astrocyte polarization of astrocytes towards the infarct core was increased after OPN treatment, possibly supporting the containment of inflammation. These findings support recent data on lacking impaired astrocyte polarization in OPN-deprived animals [5].

Our data suggest that OPN constitutes a candidate molecule to study the interaction of microglia and endogenous stem cells after stroke and could be a suitable modulating agent in CNS to support repair processes in the CNS.

## 5. Conclusion

Microglial activation and M1 and M2 polarization constitute microglia subpopulations that occur in distinct but overlapping spatial patterns following permanent focal ischemia. Osteopontin drives microglia polarization towards M2 and may be involved in locally separating M1 and M2 subpopulations locally. Whether this separation contributes to the beneficial effects of osteopontin warrants further investigation. Future studies should be directed at examining the molecular pathways linking OPN and microglial activation and focus on modifying the M2 response.

## Figures and Tables

**Table 1:** Details of primary antibodies.

Antibody	Antigene	Dilution/fixation	Identified cell type	Reference, company
Anti-NeuN	Neuronal nuclear antigen (DNA-binding)	1 : 2000 4% PFA, (2 h)	Surviving neurons	Anti-NeuN monoclonal antibody, mouse, catalog number MAB377, Merck Millipore
Anti-Iba1	Ionized calcium binding adaptor molecule 1	1 : 1000, 4% PFA, (2 h)	Activated microglia/macrophages	Anti-iba1 polyclonal antibody, rabbit, catalog number 019-19741, Wako Chemicals
Anti-iNOS	Inducible NO-synthetase	1 : 100, 4% PFA, (15 min)	M1-polarized microglia	Anti-iNOS polyclonal antibody, rabbit, catalog number ab15323, abcam
Anti-Arg1	Arginase 1	1 : 1000 4% PFA, (15 min)	M2-polarized microglia	Anti-liver-arginase polyclonal antibody, goat, catalog number ab60176, abcam
Anti-CD206	Mannose receptor	1 : 1500, 4% PFA, (30 min)	M2-polarized microglia	Anti-CD206 polyclonal antibody, goat, catalog number AF2535, R&D Systems
Anti-Ym1	Chitinase-3-like-3	1 : 100, 50% acetone (15 min, 4°C) 0.3% TX	M2-polarized microglia	Anti-Ym1 polyclonal, rabbit, catalog number 01404, Stemcell Technologies
Anti-vWF	von Willebrand factor	1 : 1500, 4% PFA, (15 min)	Endothelial cells	Anti-vWF polyclonal antibody, rabbit, catalog number ab6994, abcam
Anti-NG2	Chondroitin sulfate proteoglycan	1 : 4000, 100% acetone (10 min, −20°C)	Polydendrocytes	Anti-NG2 polyclonal antibody, rabbit, catalog number AB5320, Millipore
Anti-GFAP	Glial fibrillary acidic protein	1 : 2000, 4% PFA (overnight, 4°C)	Astrocytes	Anti-GFAP monoclonal antibody, mouse, catalog number MAB360, Millipore

## Abbreviations

Arg1: Arginase 1
CCA: Common carotid artery
DAB: Diaminobenzidine
ECM: Extracellular matrix
GFAP: Glial fibrillary acidic protein
Iba1: Ionizing calcium binding adaptor protein 1
ICA: Internal carotid artery
i.c.v.: Intracerebroventricular
IFN*γ*: Interferon *γ*
IgG: Immunoglobulin G
IL: Interleukin
iNOS: Inducible nitric oxide synthase
LPS: Lipopolysaccharide
MCAO: Middle cerebral artery occlusion
MHC: Major histocompatibility complex
NeuN: Neuronal nuclei
NG2: Chondroitin sulfate proteoglycan
NSC: Neural stem cells
NO: Nitric oxide
OPN: Osteopontin
PT: Photothrombosis
ROS: Reactive oxygen species
RNS: Reactive nitrogen species
RT: Room temperature
TGF*β*: Transforming growth factor
TNF*α*: Tumor necrosis factor *α*
vWF: von Willebrand factor
Ym1: Chitinase 3-like-3.
